# A large-scale population-based study reveals that gp42-IgG antibody is protective against EBV–associated nasopharyngeal carcinoma

**DOI:** 10.1172/JCI180216

**Published:** 2024-11-26

**Authors:** Xiang-Wei Kong, Guo-Long Bu, Hua Chen, Yu-Hua Huang, Zhiwei Liu, Yin-Feng Kang, Yan-Cheng Li, Xia Yu, Biao-Hua Wu, Zi-Qian Li, Xin-Chun Chen, Shang-Hang Xie, Dong-Feng Lin, Tong Li, Shu-Mei Yan, Run-Kun Han, Nan Huang, Qian-Yu Wang, Yan Li, Ao Zhang, Qian Zhong, Xiao-Ming Huang, Weimin Ye, Ming-Fang Ji, Yong-Lin Cai, Su-Mei Cao, Mu-Sheng Zeng

**Affiliations:** 1 State Key Laboratory of Oncology in South China, Collaborative Innovation Center for Cancer Medicine, Guangdong Key Laboratory of Nasopharyngeal Carcinoma Diagnosis and Therapy, Sun Yat-sen University Cancer Center (SYSUCC), Guangzhou, Guangdong, China.; 2 Department of Otorhinolaryngology, Sun Yat-sen Memorial Hospital, Sun Yat-sen University, Guangzhou, Guangdong, China.; 3 Zhongshan School of Medicine, Sun Yat-sen University, Guangzhou, Guangdong, China.; 4 Department of Pathology, Sun Yat-sen University Cancer Center (SYSUCC), Guangzhou, Guangdong, China.; 5 Division of Cancer Epidemiology and Genetics, National Cancer Institute, Rockville, Maryland, USA.; 6 Guangdong Provincial People’s Hospital, Guangdong Academy of Medical Sciences, Guangzhou, Guangdong, China.; 7 Guangxi Health Commission Key Laboratory of Molecular Epidemiology of Nasopharyngeal Carcinoma, Wuzhou Red Cross Hospital, Wuzhou, Guangxi, China.; 8 Department of Preventive Medicine, Wuzhou Cancer Center, Wuzhou, Guangxi, China.; 9 Cancer Research Institute of Zhongshan City, Zhongshan City People’s Hospital, Zhongshan, Guangdong, China.; 10 Key Laboratory of Carcinogenesis and Translational Research (Ministry of Education/Beijing), Laboratory of Molecular Oncology, Peking University Cancer Hospital & Institute, Beijing, China.; 11 Department of Epidemiology and Health Statistics, School of Public Health, Fujian Medical University, Fuzhou, Fujian, China.; 12 Key Laboratory of Ministry of Education for Gastrointestinal Cancer, Fujian Medical University, Fuzhou, Fujian, China.; 13 Department of Medical Epidemiology and Biostatistics, Karolinska Institutet, Stockholm, Sweden.

**Keywords:** Vaccines, Virology, Antigen, Epidemiology, Head and neck cancer

## Abstract

**BACKGROUND:**

EBV is associated with nasopharyngeal carcinoma (NPC), but the existence of a NPC protective antibody against EBV-associated antigens remains unclear.

**METHODS:**

Patients with NPC and matched controls were identified from prospective cohorts comprising 75,481 participants in southern China. ELISA and conditional logistic regression were applied to assess the effects of gp42-IgG on NPC. The expression of HLA-II, the gp42 receptor, in nasopharyngeal atypical dysplasia and its effect on EBV infection of epithelial cells were evaluated.

**FINDINGS:**

gp42-IgG titers were significantly lower in patients with NPC compared with controls across various follow-up years before NPC diagnosis (*P* < 0.05). Individuals in the highest quartile for gp42-IgG titers had a 71% NPC risk reduction compared with those in the lowest quartile (ORs_Q4vsQ1_= 0.29, 95% CIs = 0·15 to 0.55, *P* < 0.001). Each unit antibody titer increase was associated with a 34% lower risk of NPC (OR = 0.66, 95% CI = 0.54–0.81, *P*_trend_< 0.001). The protective effect of of gp42-IgG was observed in patients diagnosed 5 years or more, 1–5 years, and less than 1 year after blood collection (*P* < 0.05). HLA-II expression was detected in 13 of 27 specimens of nasopharyngeal atypical dysplasia, and its overexpression substantially promoted epithelial cell–origin EBV infection.

**CONCLUSION:**

Elevated EBV gp42-IgG titers can reduce NPC risk, indicating that gp42 is a potential EBV prophylactic vaccine target.

**TRIAL REGISTRATION:**

NCT00941538, NCT02501980, ChiCTR2000028776, ChiCTR2100041628.

**FUNDING:**

Noncommunicable Chronic Diseases-National Science and Technology Major Project (2023ZD0501003), National Natural Science Foundation of China (82030046, 82073625, 81860601, 82373655), Local Innovative and Research Teams Project of Guangdong Pearl River Talents Program (2019BT02Y198), and Central Financial Transfer Payment Projects of the Chinese Government, Cancer Research Grant of Zhongshan City.

## Abstract 2

Elevated EBV gp42-IgG can reduce NPC risk, indicating gp42 as a potential EBV prophylactic vaccine design target.

## Graphical abstract

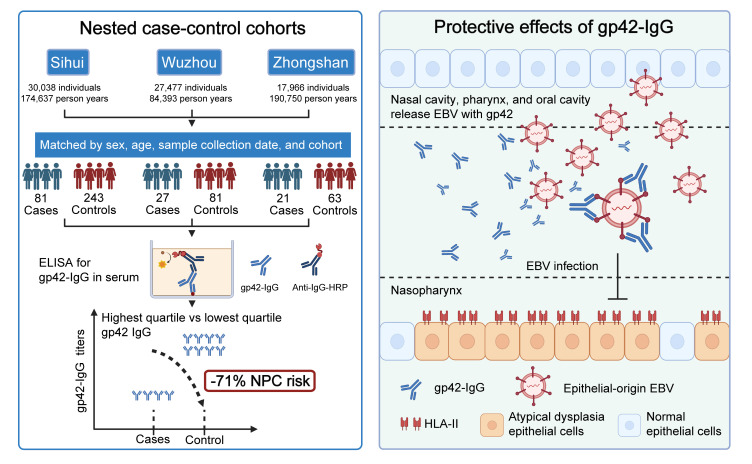

## Introduction

The incidence of nasopharyngeal carcinoma (NPC) demonstrates a striking geographical variation (1). In endemic regions such as southern China, the age-adjusted incidence can reach 23 of 100,000 in men and 7 of 100,000 in women, which is 10-fold higher than the global incidence (2). The regional variation implies a complex interplay of genetic predisposition, environmental risk factors, and, notably, EBV infection (1, 3).

In endemic regions, where more than 95% of NPC cases can be attributed to EBV infection, there is a substantial opportunity for preventative interventions through EBV vaccination to reduce NPC burden. However, a major obstacle emerges when selecting an optimal target for vaccine design, as EBV expresses as many as 5 surface glycoproteins with definitive functions (4). Glycoproteins gB, gHgL, and gp350 have been spotlighted in the development of EBV prophylactic vaccines, given their pivotal roles in EBV-host interactions (4), availability of potent monoclonal antibodies against them (5–8), and the encouraging results from preliminary laboratory-based trials (9). Nonetheless, initial studies assessing neutralizing antibodies targeting gp350 in 3 cohorts with modest sample sizes have shown inconsistent results, with 1 study showing an association with reduced NPC risk (10), while the other 2 found associations with increased NPC risk (11, 12). In our previous nested case control study of a community-based NPC screening cohort, we also tested antibodies against gp42, gHgL, gB, and gp350, as well as B cell– and epithelial cell–neutralizing antibodies. Our results suggested a potential protective role for antibodies against gp42, with gp42-IgG showing a stronger correlation with epithelial and B cell neutralization competence compared with gp42-IgA. However, the evidence remains inconclusive, partly because of the limited sample size (20 patients and 40 controls) (13). It is worth noting that vaccines aiming at gp42 have been less studied (14).

To follow-up on our initial findings, here, we conducted a larger nested case control study utilizing samples from 129 NPC patients and 387 matched controls, which were collected prospectively across 3 independent cohorts in southern China, independent from our previous study (13). Our findings confirmed the inverse association between increased levels of anti-gp42 antibodies and NPC risk. In addition, we sought to explore the physical and molecular basis by examining the presence of HLA-II, the only known receptor for gp42, in nasopharyngeal atypical dysplasia samples. This analysis showed that overexpression of HLA-II in epithelial cells promoted EBV infection. Taken together, our findings suggest that IgG antibodies against gp42 play a protective role against NPC in endemic regions, thereby marking gp42 a potential prophylactic vaccine design target to prevent NPC in high-risk populations.

## Results

### Study population.

**Figure 1:**
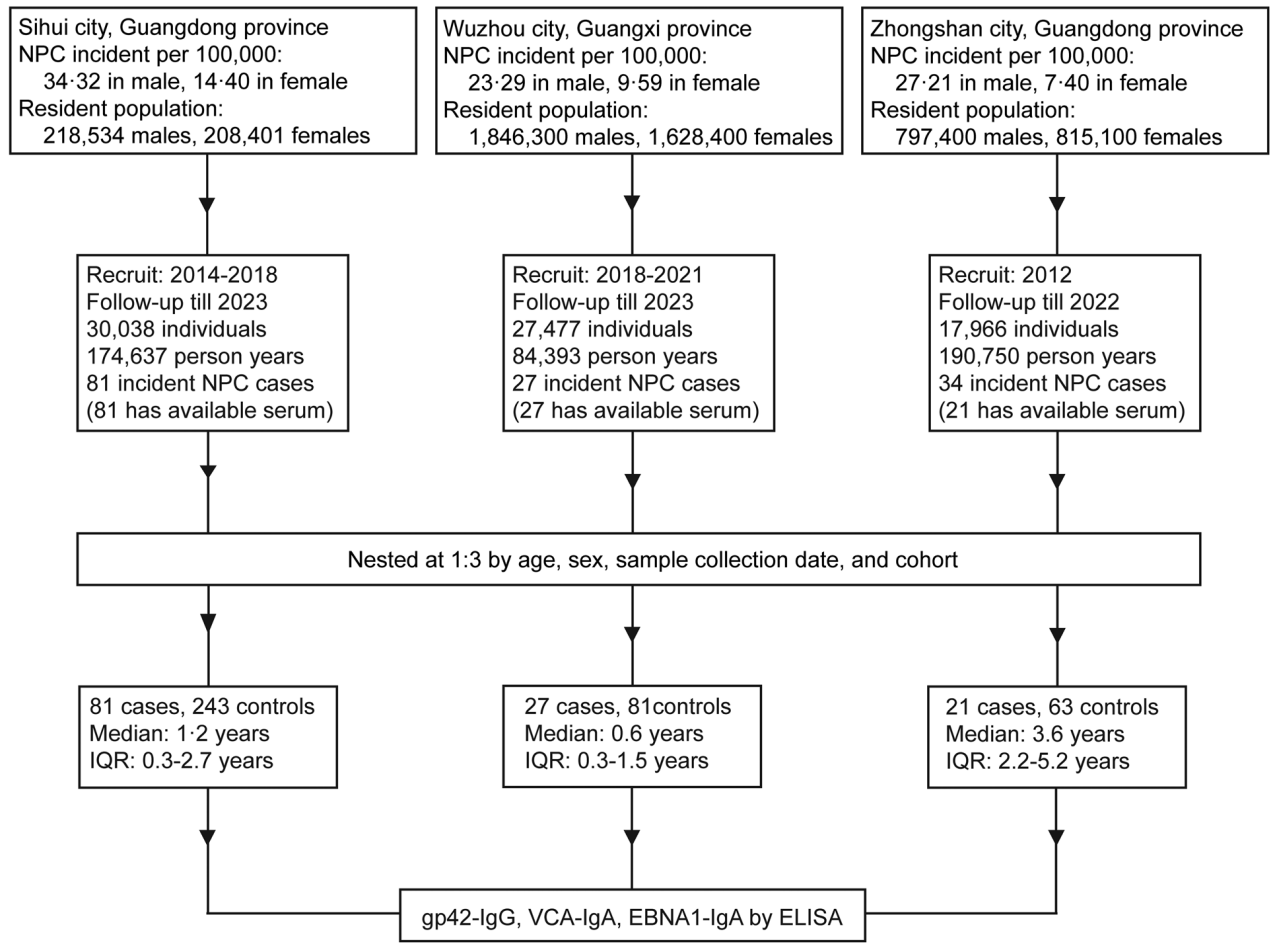
A Flow chart of the study design. A total 75,481 individuals from 3 independent prospective cohorts established in 3 NPC epidemic regions (Sihui city, Guangdong Province; Wuzhou city, Guangxi Province; Zhongshan city, Guangdong Province) in southern China were recruited from the years 2014 to 2018, 2018 to 2021, and 2012, respectively. A total of 129 serum-available incident NPC cases were identified until 2023, and each patient was matched to 3 controls from individual cohort by age, sex, and sample collection dates. Titers of gp42-IgG, VCA-IgA, and EBNA1-IgA were measured by ELISA.

Based on 3 prospective community-based cohorts among Sihui, Wuzhou, and Zhongshan cohorts with a total 75,481 individuals, we conducted a nested case control study to assess whether elevated levels of gp42-IgG are associated with a lower risk of NPC. Blood samples from 129 patients with NPC and 387 individually matched controls at a 1:3 ratio by age, sex, blood collection time and region were obtained, with a median of 1.3 years (IQR = 0.3–2.9 years) before NPC diagnosis (Figure 1). Characteristics of the patients with NPC and controls included in the analysis are shown in Table 1. A majority of participants were men (76.0%), aged 50–59 years at baseline (44.2%), and individuals with NPC were more likely to have a higher EBV antibody–based risk score compared with controls. A longer follow-up period was observed for those in the Zhongshan cohort (*P* < 0.001) (Supplemental Figure 1; supplemental material available online with this article; https://doi.org/10.1172/JCI180216DS1). All samples were independent of those from our previous study (13).

### Elevated gp42-IgG titers in disease-free controls compared with incident NPC cases.

To accurately quantify serum levels of gp42-IgG and make comparisons in different batches of samples, we developed an ELISA detection system and assessed the linearity and precision using serial dilution of monoclonal human gp42 antibody in-house. The ELISA showed excellent linearity (goodness of fit of least-squares regression *R^2^* = 0.95) over a broad dynamic range of 0.46–111 ng/mL (Supplemental Figure 2A). We demonstrated the reproducibility by repeating measurements of the 3 concentrations of monoclonal gp42 antibody standards in triplicate and across 11 batches. Results showed a coefficient of variation (CV) of less than 20% (Supplemental Figure 2B and Supplemental Table 1), with the corresponding interclass correlation coefficient (inter-CC) of 0.97 (95% CI: 0.92–0.97) and intraclass CC (intra-CC) of 0.92 (95% CI: 0.43–0.99). For the randomly selected 10% serum samples (*n* = 52), the results showed a 5% CV for the duplicated testing samples.

**Figure 2:**
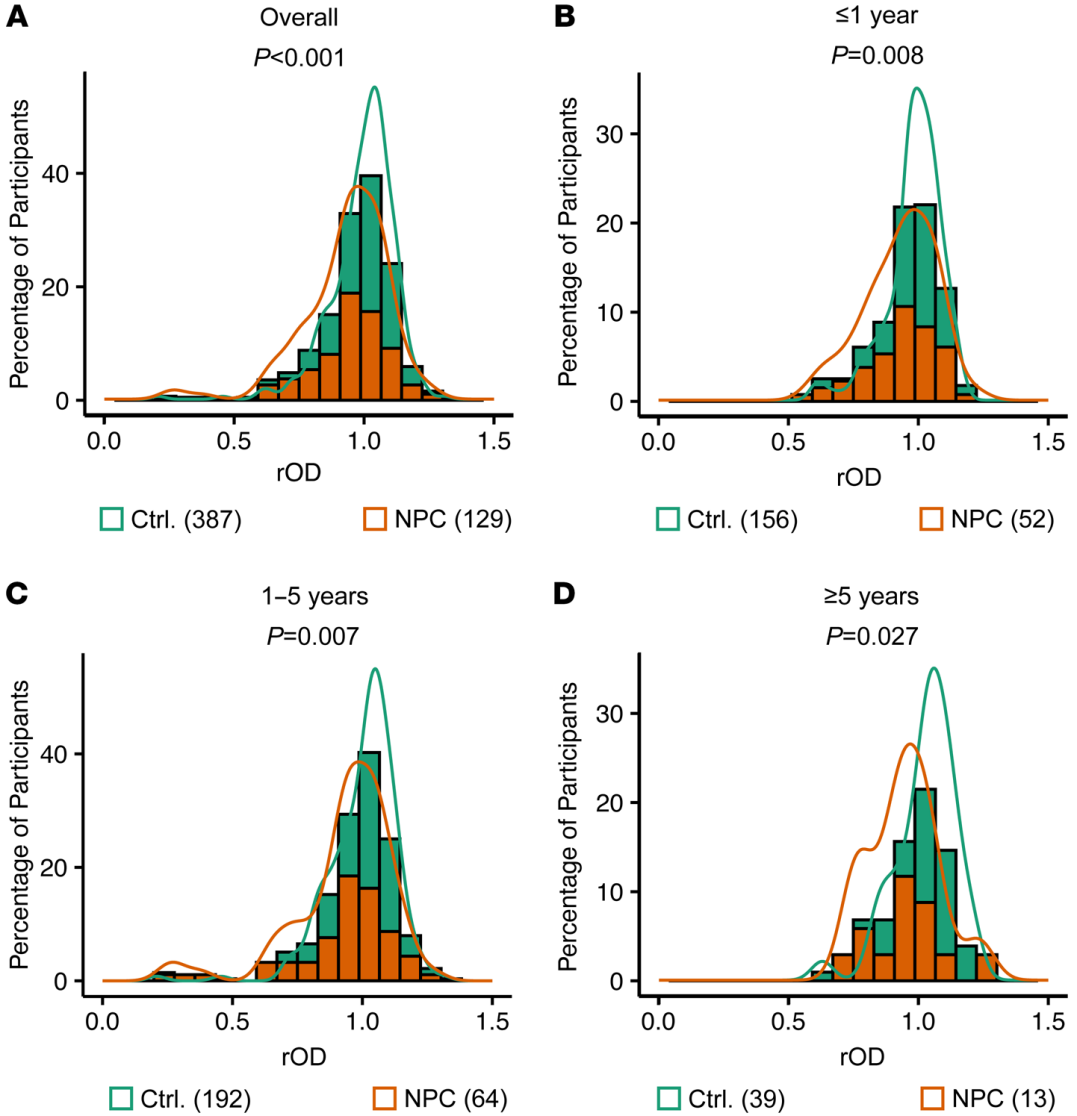
Analysis of gp42-IgG levels in the nested case control study. Distributions of gp42-IgG levels in (**A**) the overall cohort, (**B**) the cohort with a follow-up duration of 1 year or less, (**C**) the cohort with a follow-up duration of 1–5 years, and (**D**) the cohort with follow-up duration 5 or more years. rOD is a standardized OD_450_ – OD_630_ value, which was consistent across testing batches (see Methods for details). The lines at the top of each histogram represent kernel density estimations. The Wilcoxon test was used to calculate *P* values, which are shown under the subtitles. Numbers in parentheses in the legends refer to the number of participants in each group.

Notably, gp42-IgG titers were significantly lower in patients with NPC than in the controls in the overall cohort (*P* < 0.001) and in subgroups with follow-up periods of 1 year or less (*P* = 0.008), 1–5 years (*P* = 0.007), and 5 years or more (*P* = 0.027) before NPC diagnosis (Figure 2). However, the overall distribution of gp42-IgG titers remained consistent across sexes (*P* = 0.174) and age groups (*P* = 0.320) (Supplemental Figure 3). To evaluate the associations of gp42-IgG titers with the NPC risk score, we analyzed gp42-IgG titers at various EBV antibody NPC risk scores for controls according to the different follow-up durations. The result showed that the controls with a low-risk score were more likely to have higher gp42-IgG titers than did those with medium- and high-risk scores in the overall cohort (*P* < 0.001), and the same result was obtained for the subgroups with a follow-up period of 1 year or less (*P* = 0.051), 1–5 years (*P* < 0.001), and 5 years or more (*P* = 0.212) before NPC diagnosis (Supplemental Figure 4).

### The risk of NPC is highest in individuals with the lowest gp42-IgG titers.

We categorized individuals into quartiles on the basis of gp42-IgG levels in controls and explored the association between gp42-IgG levels and NPC risk using conditional logistic regression. We found that in the overall cohort, elevated levels of gp42-IgG in the highest quartile reduced NPC risk by 71% (ORs_Q4vsQ1_ = 0.29, 95% CI_Q4vsQ1_= 0.15–0.55) and that the risk of NPC decreased as the levels of gp42-IgG increased (*P*_trend_<0.001). When stratified by length of follow-up, the inverse association with a dose response trend persisted among the individuals diagnosed 1 year or less, 1–5 years, and 5 or more years after blood collection, with corresponding ORs_Q4vsQ1_ of 0.33, 0.33, and 0.05, respectively, and all *P*_trends_ of less than 0.05 (Table 2). The inverse associations could be consistently observed among the subgroups, with different sexes, ages, cohorts, and EBV antibody NPC risk scores, all with *P_heterogenity_*values were 0.05 or higher) (Supplemental Figure 5).

### Expression of HLA-II, the sole identified gp42 receptor, is detectable in nasopharyngeal atypical dysplasia.

**Figure 3:**
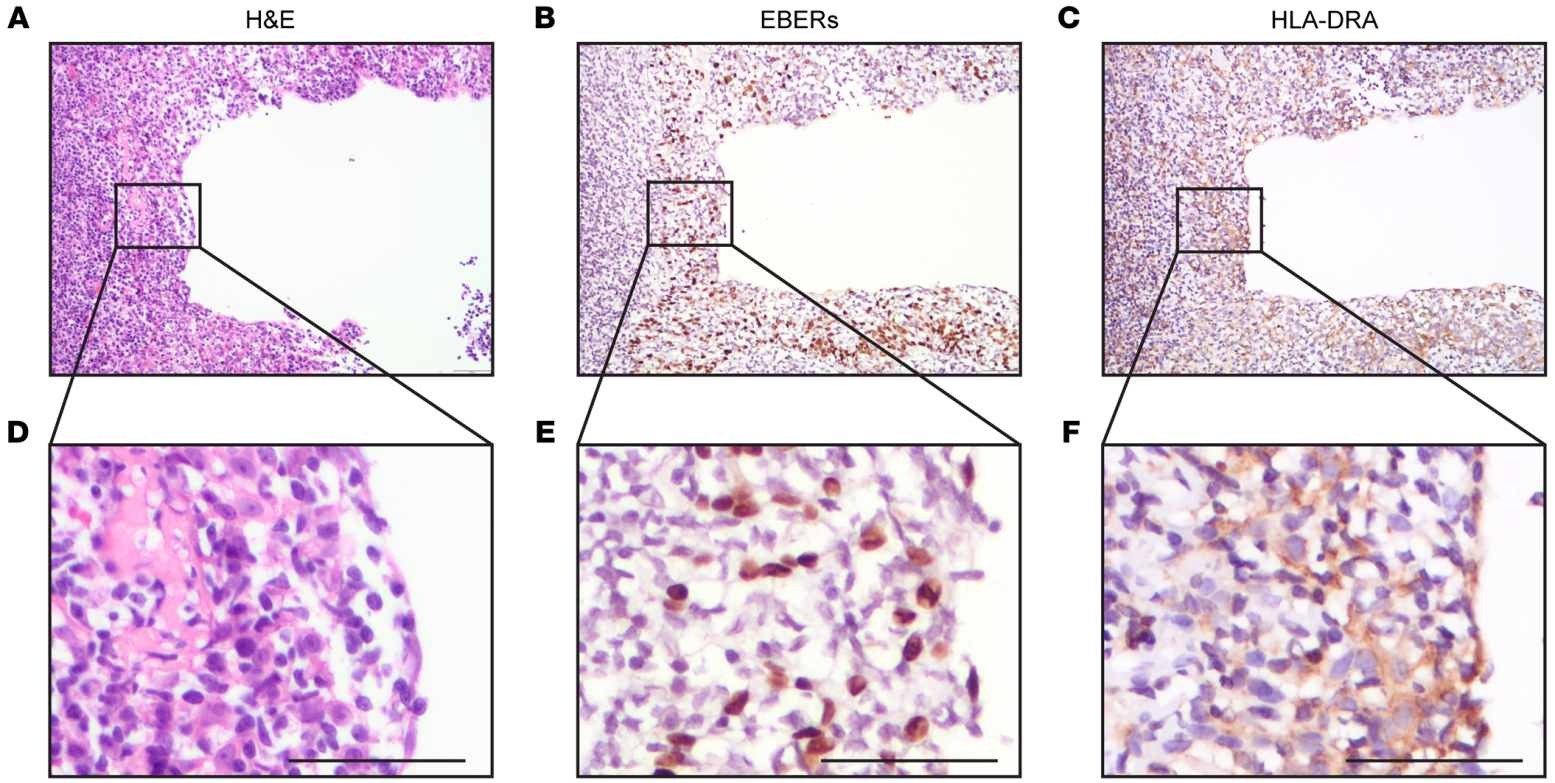
HLA-II expression in nasopharyngeal atypical dysplasia. (**A** and **D**) H&E stained images. (**B** and **E**) ISH was performed to detect EBERs. (**C** and **F**) IHC was performed to detect HLA-DRA. The sample shown is representative of 27 samples. Original magnification, ×20 (**A**–**C**). Images in **D**–**F** were zoomed in from the black outlined regions; scale bars: 50 μm.

The expression of HLA-II is notably detectable in NPC tumor cells (15); however, it remains unclear whether the expression of HLA-II precedes tumor occurrence. To address this hypothesis, we collected 27 nasopharyngeal premalignant tissues from the SYSUCC, where samples were examined by 2 experienced pathologists independently. Among the 27 premalignant samples, 20 were positive for EBV-encoded small RNAs (EBERs, 13 were positive for HLA-DRA (Major Histocompatibility Complex, Class II, DR Alpha), and 12 were positive for both HLA-DRA and EBERs (Supplemental Table 2, Figure 3, and Supplemental Figure 6).

### HLA-II facilitates epithelium-origin EBV infection of epithelial cells.

To further investigate the functional implications of HLA-II expression in epithelial cells, we established stable HEK293 and NP69 cell lines overexpressing HLA-DRA/-DRB (Major Histocompatibility Complex, Class II, DR Beta). We found that overexpression of HLA-DR notably enhanced the infection of CNE2-EBV (EBV producing from the CNE2 cell line), the representative of EBV originating from epithelial cells (Supplemental Figure 7, A and C, and Supplemental Figure 8, A and C). However, this effect was only marginal in promoting the infection of Akata-EBV, the representative of EBV originating from B cells (Supplemental Figure 7, B and D, and Supplemental Figure 8, B and D).

To further validate the role of gp42 in mediating CNE2-EBV infection, we purified soluble gp42 and HLA-DRA/-DRB. Treatment of cells with soluble gp42 and HLA-DRA/-DRB led to a dose-dependent inhibition of EBV infection (Supplemental Figure 9, A and B). Interestingly, the inhibitory effects were countered by the overexpression of HLA-DRA/-DRB (Supplemental Figure 7, E and F, and Supplemental Figure 9, C and D). Overexpression of HLA-DRA/-DRB was confirmed by Western blot analysis (Supplemental Figure 7G). These results strongly suggest that gp42 plays an important role in promoting epithelial-cell-origin EBV to infect the HLA-DR–expressing epithelial cells.

## Discussion

To our knowledge, we have conducted the largest prospective epidemiological study to date, focusing on NPC-associated protective antibodies against EBV based on 3 mass screening cohorts. Our findings establish the protective capacity of gp42-IgG against NPC in endemic regions. Moreover, our research has elucidated the underlying mechanistic foundations of these protective effects. The identification of HLA-II, the sole receptor for gp42, in nasopharyngeal atypical dysplasia constitutes, in our view, a novel discovery. Our experiments have further demonstrated that the overexpression of HLA-II amplified the infectivity of EBV within epithelial cells. Our results for the interplay between gp42-IgG, HLA-II, and EBV infection offer valuable insights into the mechanisms of the observed gp42-IgG protective effects against NPC.

Previous studies have shown that the elevation of antibodies against EBV EA, EBNA1, and VCA are associated with a higher risk of NPC, which leads to utilization the EBV-associated antibodies as the screening markers for NPC in endemic regions (16). In comparison with these markers, the precise roles of antibodies against glycoproteins in the development of NPC remain unclear. Although gB, gHgL, and gp350 play essential roles in EBV infection, antibodies targeting these components do not exhibit significant protective effects against NPC development, as demonstrated in previous studies (11–13). In this study, which included 129 incident NPC cases and 387 matched controls, we established a statistically significant reduction in NPC risk among individuals overall and among those diagnosed 1 year or less, 1–5 years, and more than 5 years after blood collection, with dose response trends. Moreover, the protective effects of gp42-IgG could be observed across both sexes, different age groups, and cohorts, indicating the robustness of the protective influence of gp42-IgG against NPC. However, in the previous report, gp42-IgG levels were nominally higher in controls than in those who developed NPC more than 5 years after blood ample collection, but lower in controls than in individuals who developed NPC within 5 years (12). This discrepancy could be due to the differences in antigenicity and measurement methods. With regard to antigenicity, our purified gp42 could substantially block EBV entry into epithelial cells (Supplemental Figure 7F and Supplemental Figure 9, B and D), which was not tested previously (12), and our purified gp42 was used to identify 2 potent EBV-neutralizing antibodies (17). As for the measurement methods, we applied ELISA versus the high-throughput Luminex assay described in the previous report (12).

Notably, gp42 is recognized as a crucial molecule that dictates the host preference for EBV (18). A higher presence of gp42, released through epithelial cell lytic infection, results in a preference for B cell infection due to the expression of HLA-II by B cells rather than epithelial cells under physiological conditions. Our research shows that the overexpression of HLA-II only minimally promoted the infection of B cell–origin EBV in epithelial cells. This observation prompted us to speculate whether, in physiological settings, while antibodies against gB, gHgL, and gp350 confer a foundational level of humoral protection against EBV infection and reactivation, the continuous exposure of epithelial cell–origin EBV in nasopharyngeal niches might require higher titers of gp42 antibodies. Such elevated titers could counteract the susceptibility of nasopharyngeal premalignant lesions to epithelial cell–origin EBV infection, thereby diminishing the risk of NPC development.

Direct evidence concerning the origin of EBV driving NPC remains elusive. Nevertheless, recent studies indicate persistent exposure to epithelial cell–origin EBV in nasopharyngeal niches. First, with regard to the Cp methylation percentage of the EBV genome, methylation levels are notably lower in lytic infection than in latent infection (19); furthermore, evidence suggests that EBV DNA can be detected in nasopharyngeal swabs as early as 3 years before NPC development (20). When compared with healthy controls, nasopharyngeal swabs from patients with NPC exhibit significantly higher Cp methylation percentages of EBV DNA (21). Second, considering gp42, the quantity of surface-bound gp42 on EBV serves as an indirect indicator of its origin. HLA-II expressed in B cells can capture and reduce the amount of surface-bound gp42 on released EBV (18). Evidence demonstrates that saliva-origin EBV has significantly higher levels of gp42 when compared with EBV originating from lymphoblastoid cell lines (LCLs) (22). Third, with respect to the EBV release rate, studies have proposed that the primary source of EBV in saliva is epithelial lytic infection, as the release rate far surpasses that of the maximum release rate from the pharyngeal lymphatic ring (23). Last, in immune-competent healthy populations, EBV lytic infection can be detected in the parotid gland and the tongue’s edge (24, 25). Collectively, these findings suggest constant exposure of epithelium-origin EBV in nasopharyngeal niches. Consequently, elevated levels of antibodies against gp42 hold the potential to reduce the likelihood of EBV infection within these niches. In our recent study, we identified 2 human anti-gp42 monoclonal antibodies, named 2C1 and 2B7, that have a strong neutralizing effect against epithelial cell–origin EBV infection of epitheliausing the NOK cell line (IC_50_ = 0.091 and 0.022 μg/mL, respectively), providing further strong evidence of the protective effects of gp42-IgG against NPC (17).

Although we have provided compelling evidence of the role of gp42-IgG in protecting against NPC, some limitations require further exploration. First, the median observation period was 1.3 years, indicating a possible reverse causality, but the fact that our results still demonstrated a significant NPC risk reduction in the individuals with a follow-up period of 5 or more years after blood collection should alleviate this reverse causality concern. Second, the protective effects on gp42-IgG in less prevalent regions remain elusive, and the correlation between gp42-IgG and other NPC risk factors, such as genetic predisposition and environmental factors, requires further exploration (1, 3). Finally, while we have proposed that HLA-II expression in premalignant nasopharyngeal dysplasia may account for the protective role of gp42-IgG, it is still unclear how gp42-IgG levels relate to HLA-II expression levels in nasopharyngeal tissue, when and how HLA-II expression is initiated, and how EBV infection in HLA-II–expressing premalignant nasopharyngeal dysplasia leads to malignant transformation.

In conclusion, we posit that the expression of HLA-II in nasopharyngeal atypical dysplasia predisposes individuals to EBV infection originating from the nasal cavity, pharynx, and oral cavity. This susceptibility can be efficiently countered by high levels of gp42-IgG, decreasing the risk of NPC development. Accordingly, prioritizing gp42-targeting EBV vaccines in high-risk populations appears warranted to mitigate NPC risk.

## Methods

### Sex as a biological variable.

Our study examined men and women, and similar findings were reported for both sexes.

### Prospective NPC screening cohorts.

This study was nested within 3 large-scale, population-based screening trials conducted in Sihui city and Wuzhou city for NPC screening, and Zhongshan city for liver cancer screening in southern China (20, 26). Between 2014 and 2018, a total of 30,038 local residents aged 30–69 years were recruited in Sihui city and followed until 2023; between 2018 and 2021, a total of 27,477 residents aged 30–69 years were recruited in Wuzhou city and followed until 2023; and in 2012, a total of 17,966 residents aged 35–64 years were recruited in Zhongshan city and followed until 2022. In the Sihui and Wuzhou cohorts, baseline serum was tested for the screening markers of EBV VCA/EBNA1-IgA by ELISA with a commercial detection kit (EUROIMMUN and Zhongshan Bio-Tech Company). NPC risk scores for each participant were determined by the risk prediction algorithm (Logit *P* = –3.934 + 2.203 × VCA-IgA + 4.797 × EBNA1-IgA) and stratified by *P* scores (low risk, *P* < 0.65; medium risk, 0.65 ≤ *P*< 0.98; high risk, *P* ≥ 0.98) (26). For individuals with low and medium NPC risk scores, EBV antibody retesting was conducted at 5- and 1-year intervals, respectively, whereas the high-risk individuals were referred for nasopharyngeal endoscopy, with a pathological biopsy performed if suspicious lesions were found. In all 3 of the cohorts, the baseline blood components (plasma, serum, and WBCs) were separated within 4–6 hours of sample collection and stored at –80^0^C for future research. The preserved baseline serum samples were used for gp42-IgG testing for all individuals in the nested case control study and for VCA-IgA and EBNA1-IgA testing in the Zhongshan cohort. Patients with NPC were identified through the local cancer and death registration system in the Zhongshan cohort, as well as in the Sihui and Wuzhou cohorts.

The median follow-up periods were 1.2 years (IQR = 0.3–2.7 years), 0.6 years (IQR = 0.3–1.5 years), and 3.6 years (IQR = 2.2–5.2 years) in the Sihui cohort, the Wuzhou cohort, and the Zhongshan cohort, respectively, and, correspondingly, 81, 27, and 34 incident NPC cases were identified in these cohorts. However, serum samples for 13 individuals in the Zhongshan cohort were unavailable. Thus, a total of 129 incident NPC cases and 387 controls were matched at a 1:3 ratio by sex, age (<40, 40–50, 50–60, ≥60 years), sample collection date (±6 months), and regions. Race and ethnicity were not considered a variable in this study.

### Soluble protein purification.

Soluble EBV (strain M81) glycoproteins gp42 and HLA-DR were purified using the 293F protein expression system cultured with a chemically defined medium (UP1000, Union Bio) in a CO_2_ incubator shaker. Transmembrane and cytoplasmic parts of gp42 and HLA-DR were truncated for the expression of soluble proteins, signal peptides were optimized for a higher yield, and the His tag was appended to the C-terminus. Six to 7 days after transfection, supernatant was collected and purified with Ni Sepharose Excel (17371202, Cytiva). Eluate was further purified using Superdex 200 Increase 10/300 GL (28990944, Cytiva). The protein concentration was determined by the bicinchoninic acid (BCA) method (23225, Pierce, Thermo Fisher Scientific). Protein was aliquoted, frozen with liquid N_2_, and stored at –80°C for further use.

### ELISA.

Soluble proteins (100 ng) were added to 96-well plates with a high binding surface, followed by overnight incubation at 4°C. Supernatant was discarded, and 5% BSA in PBST (T stands for 0.1% Tween-20) was added to block nonspecific binding followed by a 1-hour incubation at 37°C. The supernatant was then discarded. Plates were washed once with PBST. Sera were diluted to 60-fold with 5% BSA in PBST and then added to 96-well plates followed by a 1-hour incubation at 37°C. Plates were washed 3 times with PBST. Anti–human IgG was diluted at a ratio of 1:5,000 with 5% BSA in PBST and added to each well followed by a 1-hour incubation at 37°C. Plates were washed 3 times with PBST. TMB (3,3’,5,5’-Tetramethylbenzidine) (PA107-02, TIANGEN) substances were added to each well. Reactions were developed at room temperature for 10 minutes and stopped by adding HCl diluted at 1:2. Read plates were set at OD_450_ and OD_630_. In each 96-well plate, 2 wells were set to be negative controls (NCs), where no serum was added; 7 wells contained serially diluted monoclonal gp42-IgG. Then, we derived the rOD (r stands for relative) from the following equation: [(OD_450_ – OD_630_) – average (OD_450_ – OD_630_ of NCs)]/[(OD_450_ – OD_630_ of the highest concentration monoclonal anti-gp42) – average (OD_450_ – OD_630_ of NCs)].

### Randomization and masking.

Using a random number generator, samples from patients and controls by cohort were randomly assigned to each ELISA plate in a 1:3 allocation. The testing operators were blinded to the serum’s origin, and the data were pooled by independent data monitoring staff for statistical analyses.

### Linearity and precision assessment.

Linearity was assessed using serial dilution of an in-house standard monoclonal human gp42 antibody. After serial 3-fold dilution of the antibody with the initial concentration of 1,000 ng/mL, 8 samples ranging from 0.46 ng/mL to 1,000 ng/mL were tested in triplicate and evaluated (17, 27). To assess within-plate and across-plate variation, 3 concentrations of gp42-IgG standards (1,000 ng/mL, 37 ng/mL, 12.3 ng/mL) were tested in triplicate in 1 plate and across 11 batches. Inter- and intraplate variances and agreements were assessed using the inter-CCs and intra-CCs (28). In addition, 10% of the serum samples were randomly selected for duplicate testing in each plate to further evaluate the precision of gp42-IgG. A standardized value (rOD) — the mean of duplicate testing — was used for statistical analysis.

### EBV virion preparation.

Briefly, Akata-strain EBV virions were prepared from CNE2-EBV cell strains and Akata-EBV cell strains, in which the former and the latter represented epithelium- and lymphocyte-origin EBV. Preparation of these virions followed the method described in more detail in previous reports (15).

For epithelium-origin EBV preparation, CNE2-EBV cell strains were cultured in RPMI 1640 (C11875500BT, Gibco, Thermo Fisher Scientific) supplied with 10% FBS (FSP500, Ex Cell Bio). When cells reached 80% confluence, TPA (phorbol12-myristate-13-acetate) (50601ES03, Yeasen) and sodium butyrate (NaB) (B5887-1G, MilliporeSigma) were added to induce EBV lytic replication. Twelve hours after induction, the medium was replaced with fresh RPMI 1640 supplied with 10% FBS.

As for lymphocyte-origin EBV preparation, Akata-EBV cell strains were cultured in RPMI 1640 supplemented with 5% FBS. Cells were aliquoted at a density of 2 × 10^6^/mL, then goat anti–human IgG (H0111-6-100ML, Tianfun Xinqu Zhenglong Biochem. Lab) was added to induce EBV lytic replication. Six hours after induction, the medium was replaced with fresh RPMI 1640 supplemented with 5% FBS. For both preparations, 3 days after medium replacement, the supernatant was collected, filtered, and centrifuged. Pellets that contained virions were resuspended with RPMI 1640. Resuspended virions were frozen in liquid N_2_ and then stored at –80°C for further use.

### EBV in vitro infection analysis.

Epithelial cell lines were seeded in 96-well plates. Twenty-four hours later, virions with or without the indicated concentration of sHLA-DR or soluble gp42 (sgp42) were prepared, incubated at 37°C for an hour, and then added to the cells. Twenty-four to 48 hours later, flow cytometry was performed to evaluate green fluorescence. For the IC_50_ fitting, the percentage of fluorescent cells was normalized by cells infected with virions without soluble proteins, and dilution ratios were log_10_-transformed before nonlinear regression was applied. IC_50_ values were fitted using GraphPad Prism 9 (GraphPad Software).

### ISH and IHC.

Patients with atypical dysplasia in the nasopharynx were identified through the pathological station system at the SYSUCC between January 2009 and April 2020. Samples from a total of 27 patients with atypical dysplasia in the nasopharynx with adequate formalin-fixed, paraffin-embedded (FFPE) specimens were collected for subsequent testing. The EBV probe ISH kit (ISH-7001, ZSGB-BIO) was used to detect EBERs in TMA slides, following the manufacturer’s protocol. The immunohistochemical stains were performed on a Leica Autostainer (BOND-MAX, M495644) with Bond Polymer Refine Detection Kit (Leica Biosystems Newcastle Ltd) with anti–HLA-DRA1 (diluted 1:600, catalog ab9251–100 μL, Abcam) as the primary antibody. The results were evaluated by 2 experienced pathologists who were blinded to the clinical data.

### Antibodies.

The following antibodies were used in this study: anti–HLA-DRA1 (ab92511, Abcam), anti–HLA-DRB1 (ab133578, Abcam), anti–α-tubulin (2125S, CST), anti–rabbit IgG-HRP (31460, Thermo Fisher Scientific), and anti–human IgG-HRP (ab6759, Abcam).

### Cell lines.

HEK293 and NP69 cell lines were maintained in a humidified atmosphere at 37°C with 5% CO_2_. HEK293 cells were cultured in DMEM (11965092, Gibco, Thermo Fisher Scientific) supplemented with 10% FBS (FSP500, Bio Ex Cell). NP69 cells were cultured in Keratinocyte SFM (1×) (17005042, Gibco, Thermo Fisher Scientific). Details on the establishment of the HLA-II–overexpressing cell lines are provided in our previous study (17).

### Statistics.

For analysis of differences in detected antibodies and neutralizing antibody titers, Wilcoxon or Kruskal-Wallis tests were applied for 2 or more stratifications of the characteristics. Continuous gp42-IgG titers were discretized according to the cutoffs based on the overall control groups, allowing for direct comparisons across different stratifications. Conditional logistic regression was applied for nested data analysis to estimate ORs and 95% CIs for the association between antibody and NPC risk, stratified by time between baseline sampling and NPC diagnosis (<1 year, 1–5 years, and ≥5 years). No covariate was used to adjust the OR. Categorical variables were treated as continuous variables in *P*_trend_ and *P*_heterogenity_ analyses. For EBV infection rate differences under various circumstances, the 2-tailed *t* test was adopted in cases where residues complied normal distribution. All statistical analysis was done using R (4.3.1) and R packages (forestplot_3.1.1, survminer_0.4.9, survival_3.5-520). A *P* value of less than 0.05 was considered significant. Data are presented as the mean ± SEM.

### Study approval.

This study has been conducted in accordance with the Declaration of Helsinki, and the trial protocols were approved by the Ethics Review Committee of Sun Yat-sen University Cancer Center (YP2009051, B2020-362-01), Wuzhou Red Cross Hospital (LL2017-19), and Zhongshan City People’s Hospital [ZSKY2012(02)]. This study is registered with ClinicalTrials.gov (NCT00941538, NCT02501980) and the Chinese Clinical Trial Registry (ChiCTR2000028776, ChiCTR2100041628). All participants signed the informed consent form upon recruitment.

### Data availability.

Values for all data points in graphs are reported in the Supporting Data Values file. Data for study are available upon reasonable request from the corresponding authors.

## Author contributions

MSZ, SMC, and XWK were responsible for conceptualization. MSZ, SMC, YCL, MFJ, and XWK were responsible for methodology. XWK, GLB, YHH, YFK, YCL, XY, BHW, ZQL, XCC, SHX, DFL, TL, SMY, RKH, NH, QYW, YL, and AZ were responsible for investigation. XWK and HC were responsible for visualization. MSZ, SMC, YLC, and MFJ were responsible for funding acquisition. MSZ, SMC, YCL, and MFJ supervised the study. XWK wrote the original draft. MSZ, SMC, YCL, MFJ, ZWL, QZ, XMH, and WY reviewed and revised the manuscript. The order of the co–first authors was assigned according to their contributions to this study.

## Supplementary Material

Supplemental data

ICMJE disclosure forms

Unedited blot and gel images

Supporting data values

## Figures and Tables

**Table 2:**
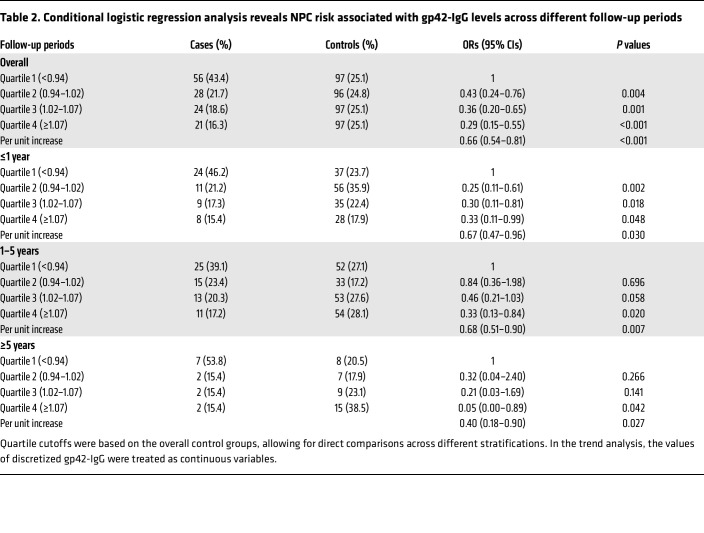
Conditional logistic regression analysis reveals NPC risk associated with gp42-IgG levels across different follow-up periods

**Table 1:**
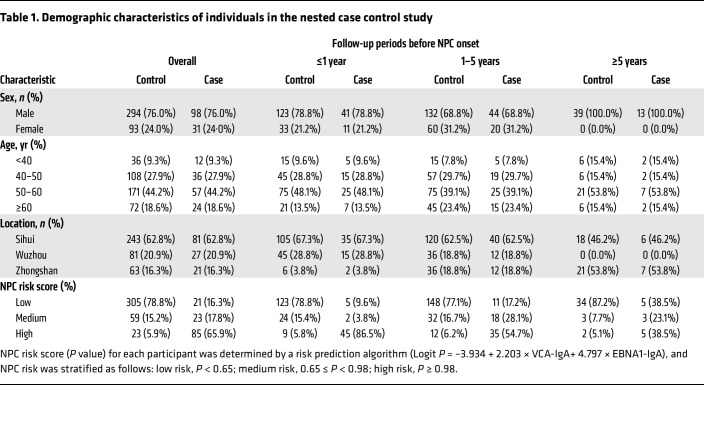
Demographic characteristics of individuals in the nested case control study

## Version 1 11/26/2024

In-Press Preview

## Version 2 02/17/2025

Electronic publication
