# Supplementary material for: A large-scale population-based study reveals that gp42-IgG antibody is protective against EBV–associated nasopharyngeal carcinoma
Source: J Clin Invest. 2024 Nov 26;135(4):e180216. doi: 10.1172/JCI180216 (PMC11827846; doi:10.1172/JCI180216)
Supplement: Supplemental data [file jci-135-180216-s210.pdf]

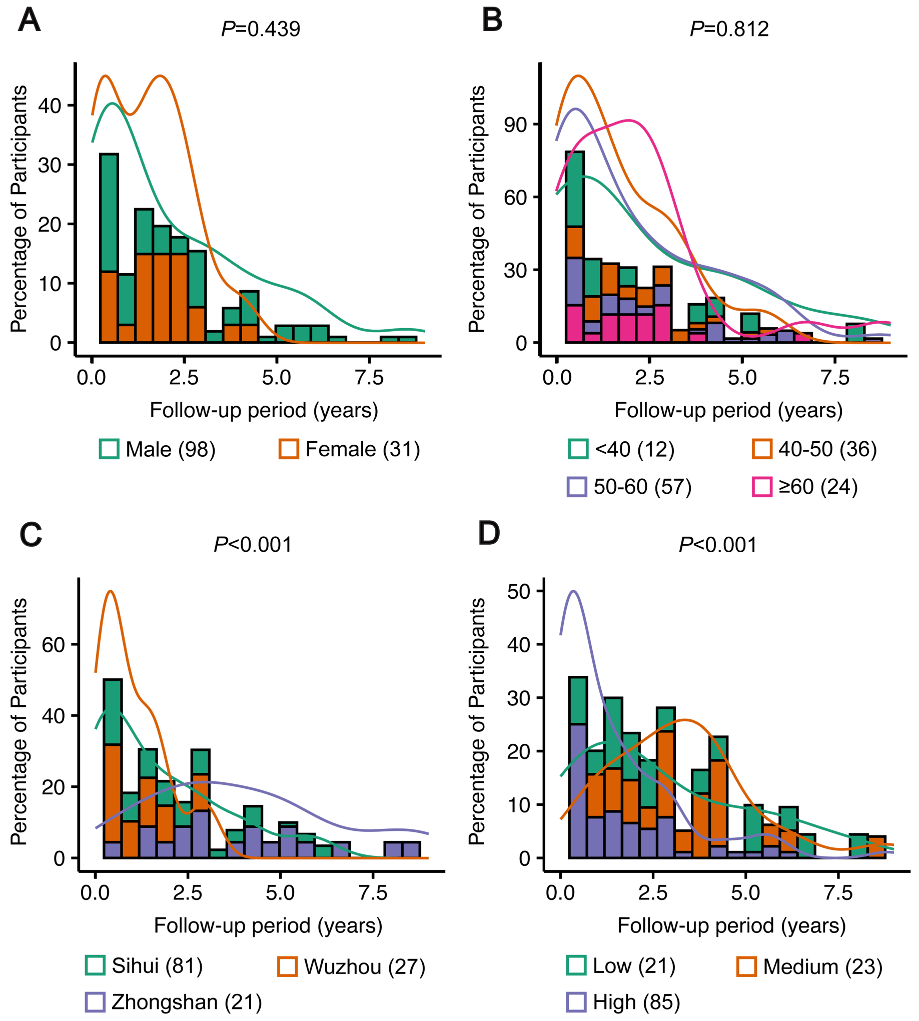

Supplemental Figure 1. Distribution of follow-up duration before nasopharyngeal carcinoma onset in the subgroups. The distribution of follow-up period before nasopharyngeal carcinoma onset stratified by sex (**A**), age (**B**), cohort (**C**), and NPC risk score (**D**). Continuous follow-up period was discretized into 20 consecutive bins. The lines at the top of each histogram represent kernel density estimations whose Y axes correspond to the density. Wilcoxon test (**A**) or Kruskal-Wallis test (**B-D**) were used to calculate *P* values. Numbers in the legends refer to the numbers of participants in each group.

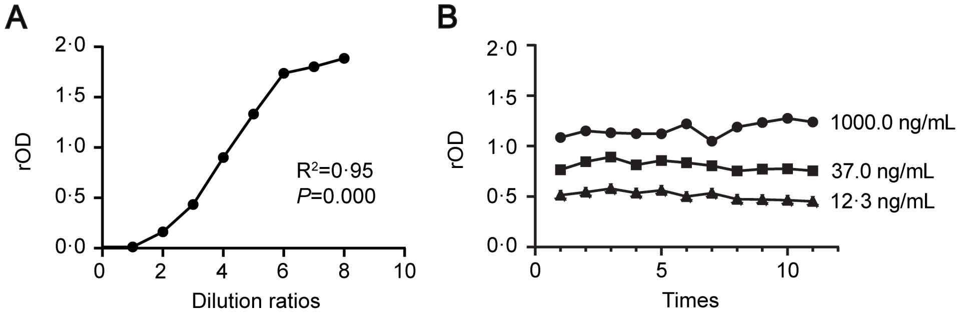

Supplemental Figure 2. Quality control testing in ELISA experiments. (**A**) The linear range was assessed using 7 samples simulated by three-fold dilution of monoclonal human gp42 (1μg/mL) mixed with negative solvent. (**B**) The rOD values of monoclonal gp42-IgG standards at three different concentrations across 11 plates.

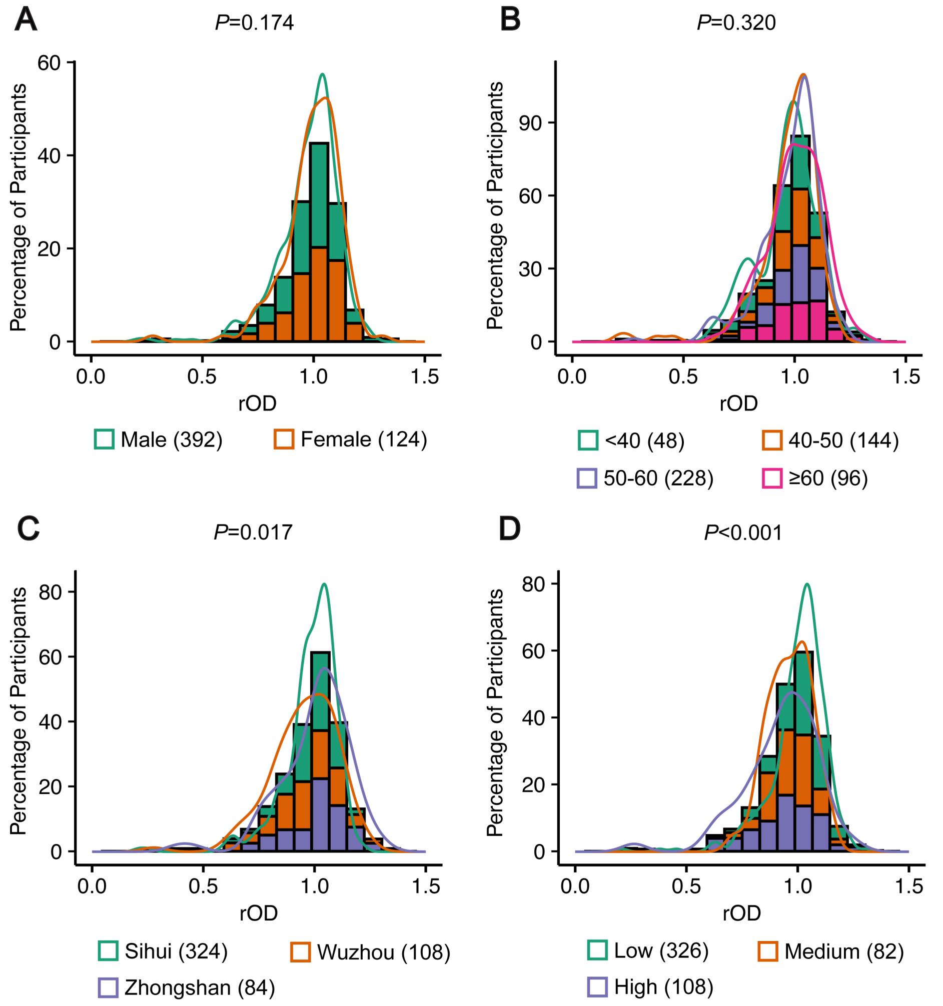

Supplemental Figuer 3. Distribution of gp42-IgG levels in the subgroups. The distributions of gp42-IgG stratified by sex (**A**), age (**B**), cohort (**C**), and NPC risk score (**D**). Continuous rOD was discretized into 20 consecutive bins. The lines at the top of each histogram represent kernel density estimations whose Y axes correspond to the density. Wilcoxon test (**A**) or Kruskal-Wallis test (**B-D**) were used to calculate *P* values. Numbers in the legends refer to the numbers of participants in each group.

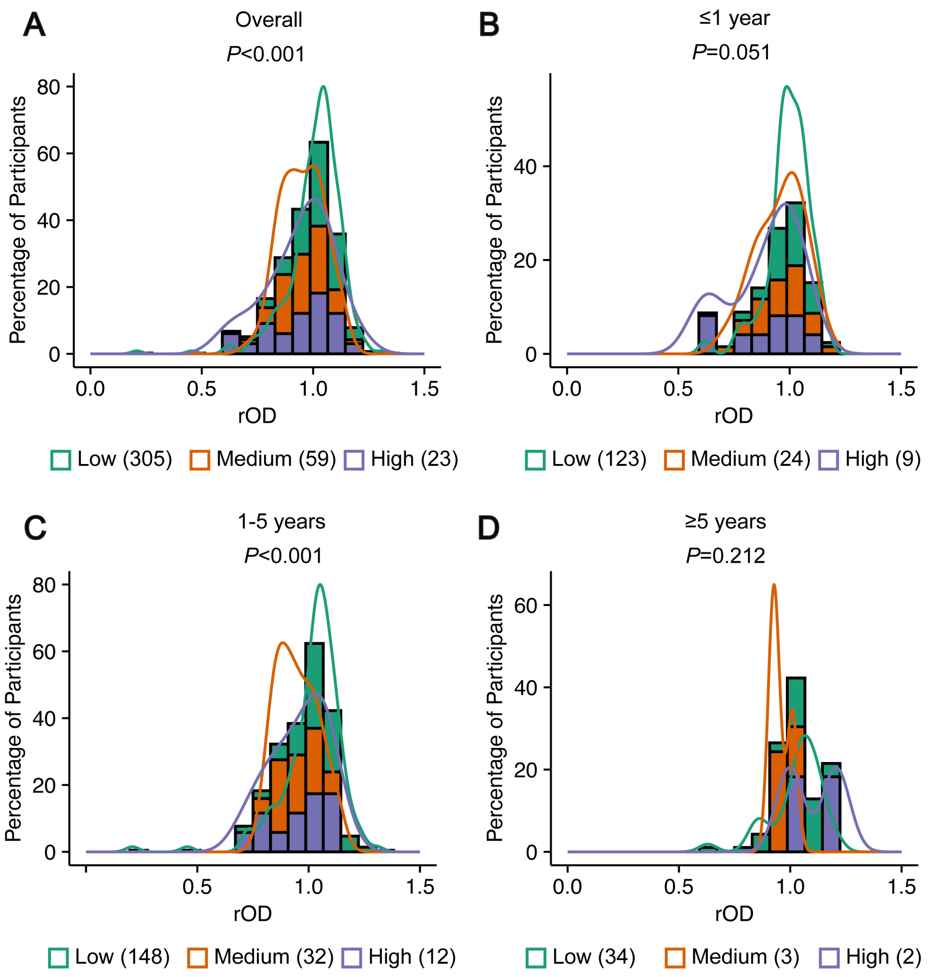

Supplemental Figure 4. Analysis of gp42-IgG titers at various EBV antibody NPC risk scores in the controls by different follow-up durations. Distributions of gp42-IgG titers in the overall cohort (**A**), the cohort with follow-up duration ≤1 year (**B**), 1-5 years (**C**), and ≥5 years (**D**). rOD was a standardized OD450-OD630 which is comparable across batches of tests. NPC risk score of each participant was determined by a risk prediction algorithm (Logit P=-3·934+2·203×VCA-IgA+4·797×EBNA1-IgA) and the individuals were stratified by the scores (Low risk, P<0·65; Medium risk, 0·65≤P<0·98; High risk, P≥0·98) . Continuous rOD was discretized into 20 consecutive bins. The lines at the top of each histogram represent kernel

density estimations whose Y axes correspond to the density. Kruskal-Wallis test were used to calculate *P* values. Numbers in the legends refer to the numbers of participants in each group.

Variables	Cases (%)	Controls (%)	*P* values
**Cohorts**			$P_{heterogeneity}$=0.150
**Sihui**			
**Quartile 1 (<0.94)**	36 (44.4)	49 (20.2)	
**Quartile 2 (0.94–1.02)**	18 (22.2)	72 (29.6)	0.001
**Quartile 3 (1.02–1.07)**	17 (21)	65 (26.7)	0.002
**Quartile 4 (≥1.07)**	10 (12.3)	57 (23.5)	<0.001
**Wuzhou**			
**Quartile 1 (<0.94)**	13 (48.1)	31 (38.3)	
**Quartile 2 (0.94–1.02)**	7 (25.9)	13 (16)	0.913
**Quartile 3 (1.02–1.07)**	3 (11.1)	19 (23.5)	0.105
**Quartile 4 (≥1.07)**	4 (14.8)	18 (22.2)	0.167
**Zhongshan**			
**Quartile 1 (<0.94)**	7 (33.3)	17 (27)	
**Quartile 2 (0.94–1.02)**	3 (14.3)	11 (17.5)	0.573
**Quartile 3 (1.02–1.07)**	4 (19)	13 (20.6)	0.667
**Quartile 4 (≥1.07)**	7 (33.3)	22 (34.9)	0.635
**Sex**			$P_{heterogeneity}$=0.427
**Male**			
**Quartile 1 (<0.94)**	42 (42.9)	79 (26.9)	
**Quartile 2 (0.94–1.02)**	21 (21.4)	72 (24.5)	0.031
**Quartile 3 (1.02–1.07)**	21 (21.4)	75 (25.5)	0.021
**Quartile 4 (≥1.07)**	14 (14.3)	68 (23.1)	0.003
**Female**			
**Quartile 1 (<0.94)**	14 (45.2)	18 (19.4)	
**Quartile 2 (0.94–1.02)**	7 (22.6)	24 (25.8)	0.024
**Quartile 3 (1.02–1.07)**	3 (9.7)	22 (23.7)	0.004
**Quartile 4 (≥1.07)**	7 (22.6)	29 (31.2)	0.008
**Age**			$P_{heterogeneity}$=0.912
**Age<50**			
**Quartile 1 (<0.94)**	22 (45.8)	35 (24.3)	
**Quartile 2 (0.94–1.02)**	10 (20.8)	45 (31.2)	0.011
**Quartile 3 (1.02–1.07)**	9 (18.8)	34 (23.6)	0.039
**Quartile 4 (≥1.07)**	7 (14.6)	30 (20.8)	0.024
**Age>=50**			
**Quartile 1 (<0.94)**	34 (42)	62 (25.5)	
**Quartile 2 (0.94–1.02)**	18 (22.2)	51 (21)	0.109
**Quartile 3 (1.02–1.07)**	15 (18.5)	63 (25.9)	0.008
**Quartile 4 (≥1.07)**	14 (17.3)	67 (27.6)	0.003
**NPC risk score**			$P_{heterogeneity}$=0.946
**Low and medium**			
**Quartile 1 (<0.94)**	17 (38.6)	88 (24.2)	
**Quartile 2 (0.94–1.02)**	9 (20.5)	90 (24.7)	0.078
**Quartile 3 (1.02–1.07)**	13 (29.5)	93 (25.5)	0.277
**Quartile 4 (≥1.07)**	5 (11.4)	93 (25.5)	0.010
**High**			
**Quartile 1 (<0.94)**	39 (45.9)	9 (39.1)	
**Quartile 2 (0.94–1.02)**	19 (22.4)	6 (26.1)	0.370
**Quartile 3 (1.02–1.07)**	11 (12.9)	4 (17.4)	0.361
**Quartile 4 (≥1.07)**	16 (18.8)	4 (17.4)	0.298

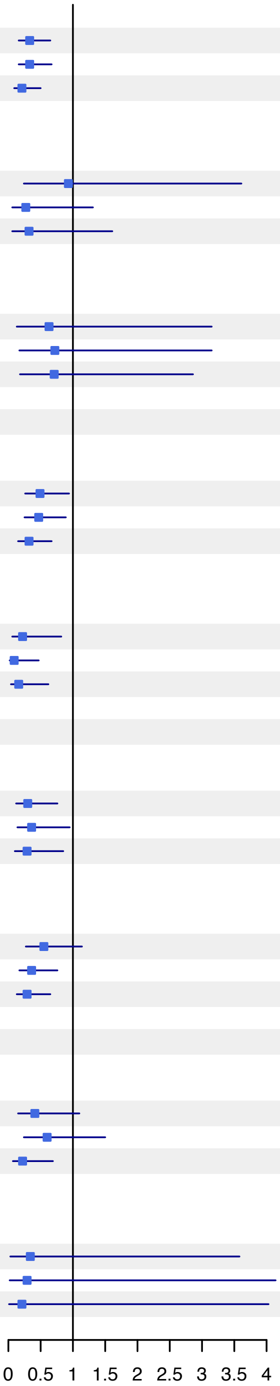

Supplemental Figure 5. Conditional logistic regression analysis reveals the risks of nasopharyngeal carcinoma in the stratified populations with various characteristics. Each square represents the point estimation of OR, the vertical bar represents the 95% CIs of ORs**.**

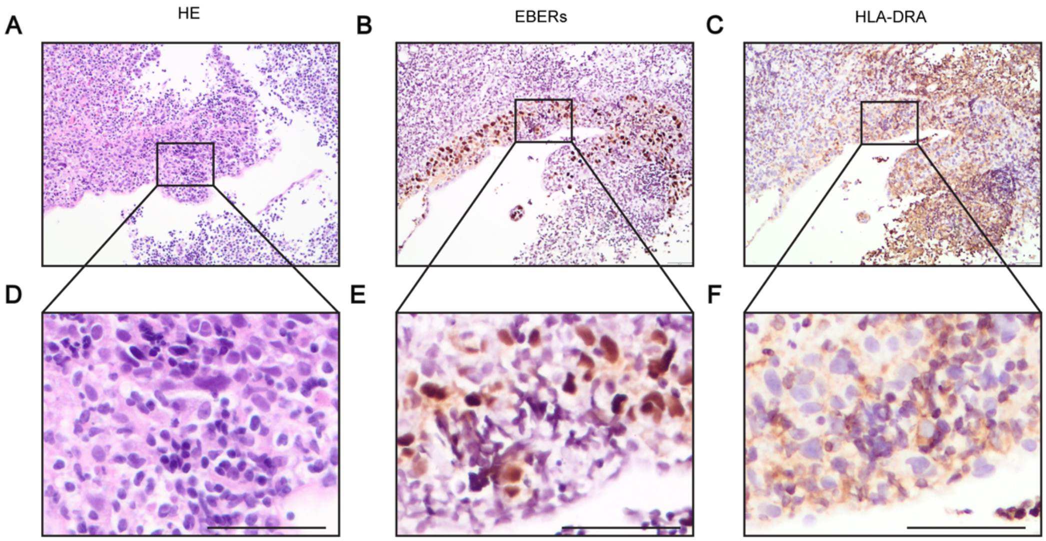

Supplemental Figure 6. HLA-II expression in nasopharyngeal atypical dysplasia. (**A** and **D**) HE staining. (**B** and **E**) In situ hybridization to detect EBERs. (**C** and **F**) Immunohistochemistry to detect HLA-DRA. Shown was a representative out of 27 samples. **A**-**C** were shot at 20×, **D**-**F** were zoomed in from the black square region, and scale bars at lower right represent 50 μm.

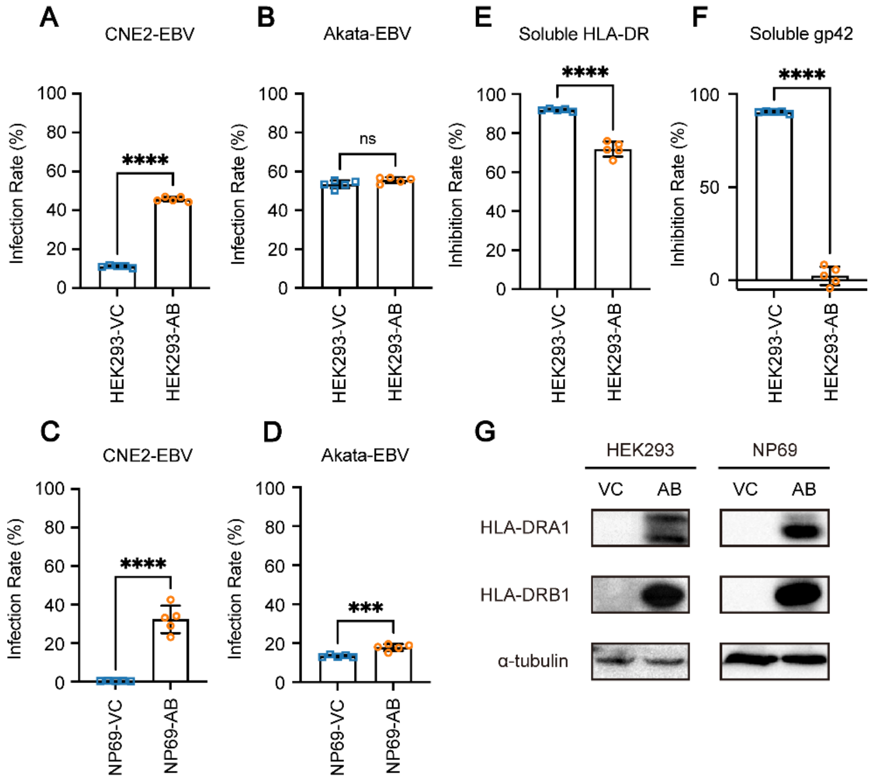

Supplemental Figure 7. Overexpression of HLA-II promotes epithelia origin EBV infection. We have established four cell lines called HEK293-VC, HEK293-AB, NP69-VC, and NP69-AB that stably transfected with either pLVX or pLVX-HLA-DRA1/DRB1. (**A** and **B**) CNE2-EBV (**A**) or Akata-EBV (**B**) infected HEK293-VC, HEK293-AB. (**C** and **D**) CNE2-EBV (**C**) or Akata-EBV (**D**) infected NP69-VC, NP69-AB. (**E**) Soluble HLA-DR at 100 μg/mL inhibited CNE2-EBV infecting HEK293-VC and HEK293-AB. (**F**) Soluble gp42 at 0·39 μg/mL inhibited CNE2-EBV infecting HEK293-VC and HEK293-AB. Inhibition rate was calculated by subtracting the relative infection rate from 100%. Each bar represents the mean of infection rate or inhibition rate. Error bars represent the SD deviation from the mean. Each group consisted of 5 samples. ns: not significant; ***: $P<0·001$; ****: $P<0·0001$. t-test was adopted. Residues complied normal distribution. (**G**) Verification of HLA-DRA and HLA-DRB expression by WB.

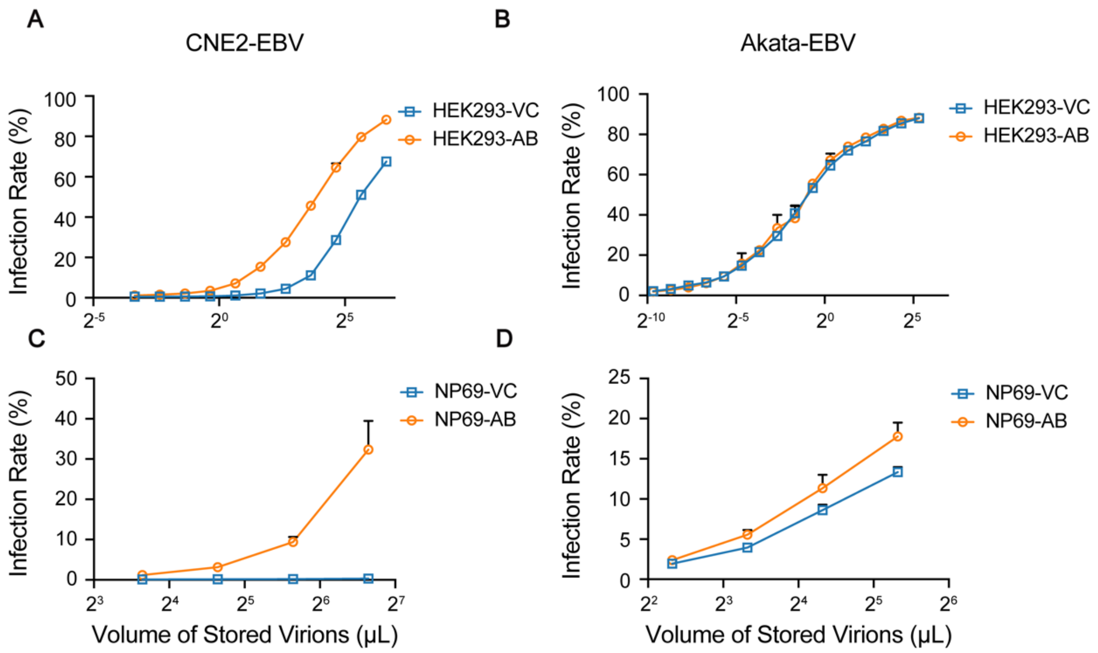

Supplemental Figure 8. Kinetics of EBV infection in various cell lines. HEK293-VC, HEK293-AB, NP69-VC, and NP69-AB that stably transfected with either pLVX or pLVX-HLA-DRA1/DRB1 were establishes. (**A** and **B**) Various volume of CNE2-EBV (**A**) or Akata-EBV (**B**) infects HEK293-VC or HEK293-AB. (**C** and **D**) Various volume of CNE2-EBV (**C**) or Akata-EBV (**D**) infects NP69-VC or NP69-AB. Each dot represents the mean infection rate of five replicates. Error bars represent SD. Each group consisted of 5 samples.

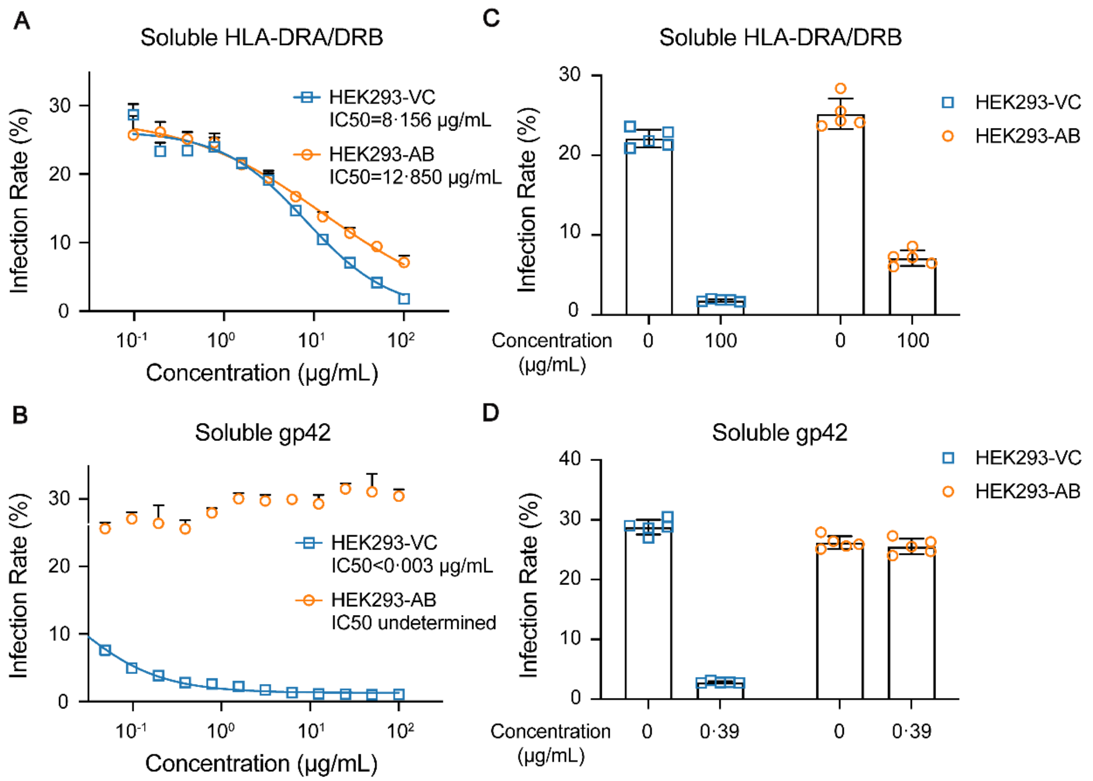

Supplemental Figure 9. Soluble HLA-DRA/DRB and gp42 significantly inhibit CNE2-EBV infecting HLA-DRA/DRB overexpressing epithelial cell lines. (**A** and **B**) CNE2-EBV Infection kinetics of two cell lines under various concentrations of soluble HLA-DRA/DRB (**A**) or soluble gp42 (**B**). Each dot represents the mean infection rate of five replicates. Error bars represent SD. (**C** and **D**) Bar plots of the infection rate of CNE2-EBV under indicated concentration of soluble HLA-DRA/DRB (**C**) or soluble gp42 (**D**). Each dot represents one test sample. The height of bars represents the mean infection rate. Error bars represent SD. Each group consisted of 5 samples.

Supplemental Table 1. Intra-plate and inter-plate variances of gp42-IgG standards

Sample[a]	Inter-plate variance (n=11)			Intra-plate variance(n=3)		
	Mean	SD	CV (%)	Mean	SD	CV (%)
1000.0 ng/mL	1·17	0.07	6·10	1.32	0.11	8.50
37.0 ng/mL	0·81	0.05	5·79	0.97	0.12	12.04
12.3 ng/mL	0·51	0.04	8·32	0.51	0.07	13.58
Overall			11.88			19.12

a. Three concentrations of the monoclonal gp42-IgG antibody standards were produced in house (ref 17)

Supplemental Table 2. Detection of EBERs in immunohistochemistry and HLA-DRA in situ hybridization of 27 atypical hyperplasia in nasopharynx.

	EBERs		
HLA-DRA	Positive	Negative	Total
Positive	12	1	13
Negative	8	6	14
Total	20	7	27
