# Supplementary material for: A large-scale population-based study reveals that gp42-IgG antibody is protective against EBV–associated nasopharyngeal carcinoma
Source: J Clin Invest. 2024 Nov 26;135(4):e180216. doi: 10.1172/JCI180216 (PMC11827846; doi:10.1172/JCI180216)
Supplement: Unedited blot and gel images [file jci-135-180216-s212.pdf]

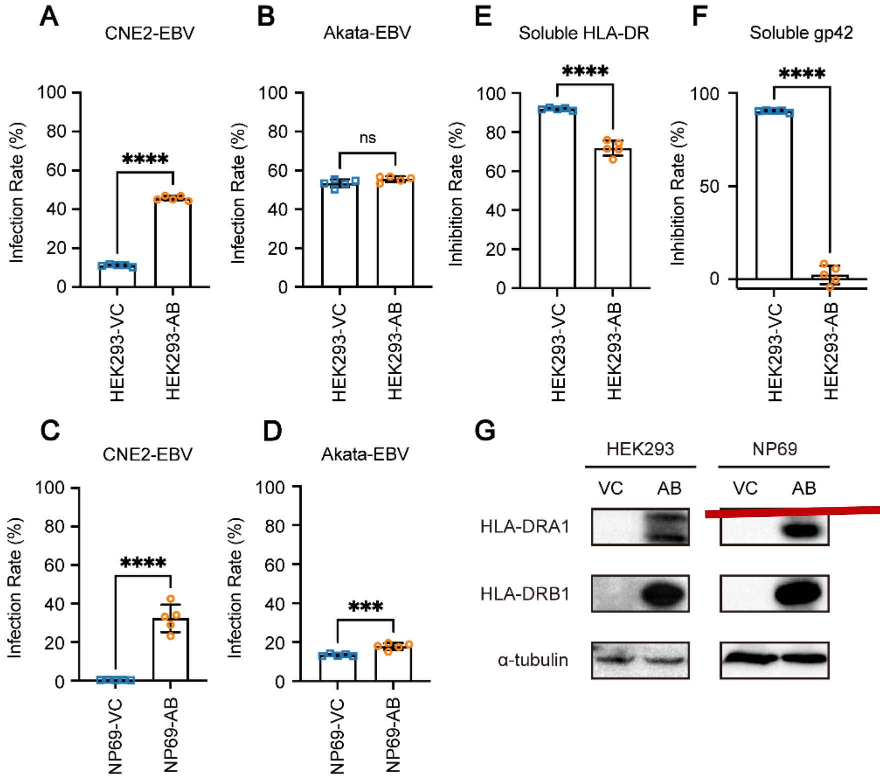

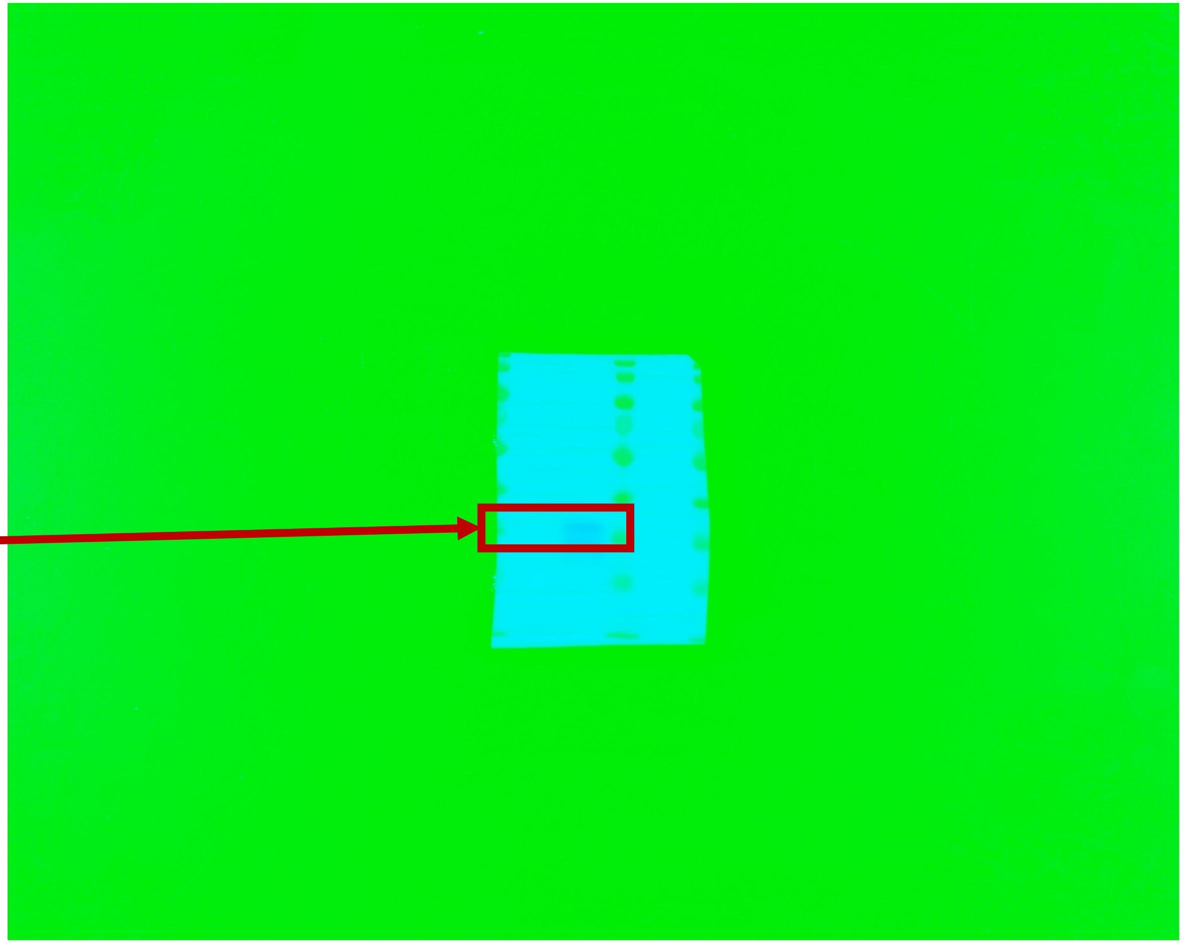

Full unedited gel for Figure 4G. Anti-HLA-DRA1 (ab92511, Abcam) and anti-rabbit IgG-HRP (31460, Thermo Fisher) were used for blotting.

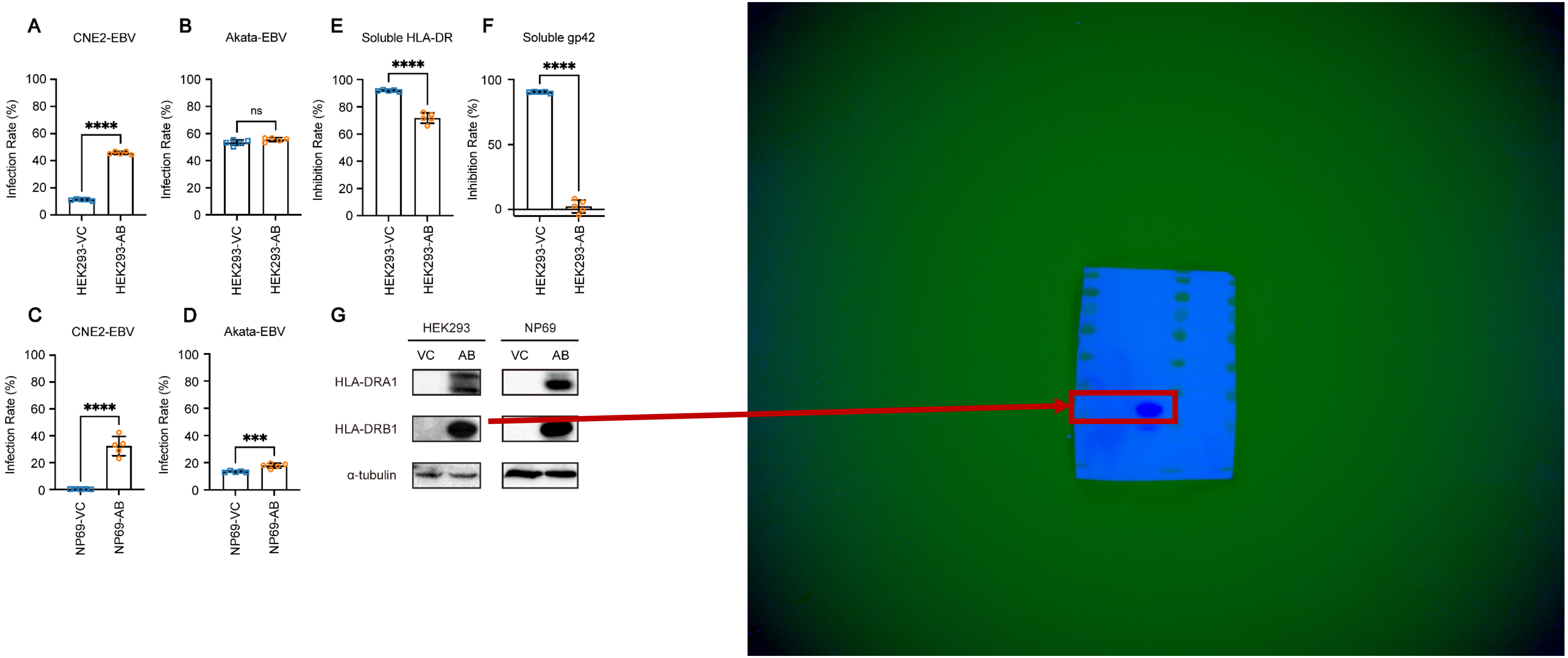

Full unedited gel for Figure 4G. Anti-HLA-DRB1 (ab133578, Abcam) and anti-rabbit IgG-HRP (31460, Thermo Fisher) were used for blotting.

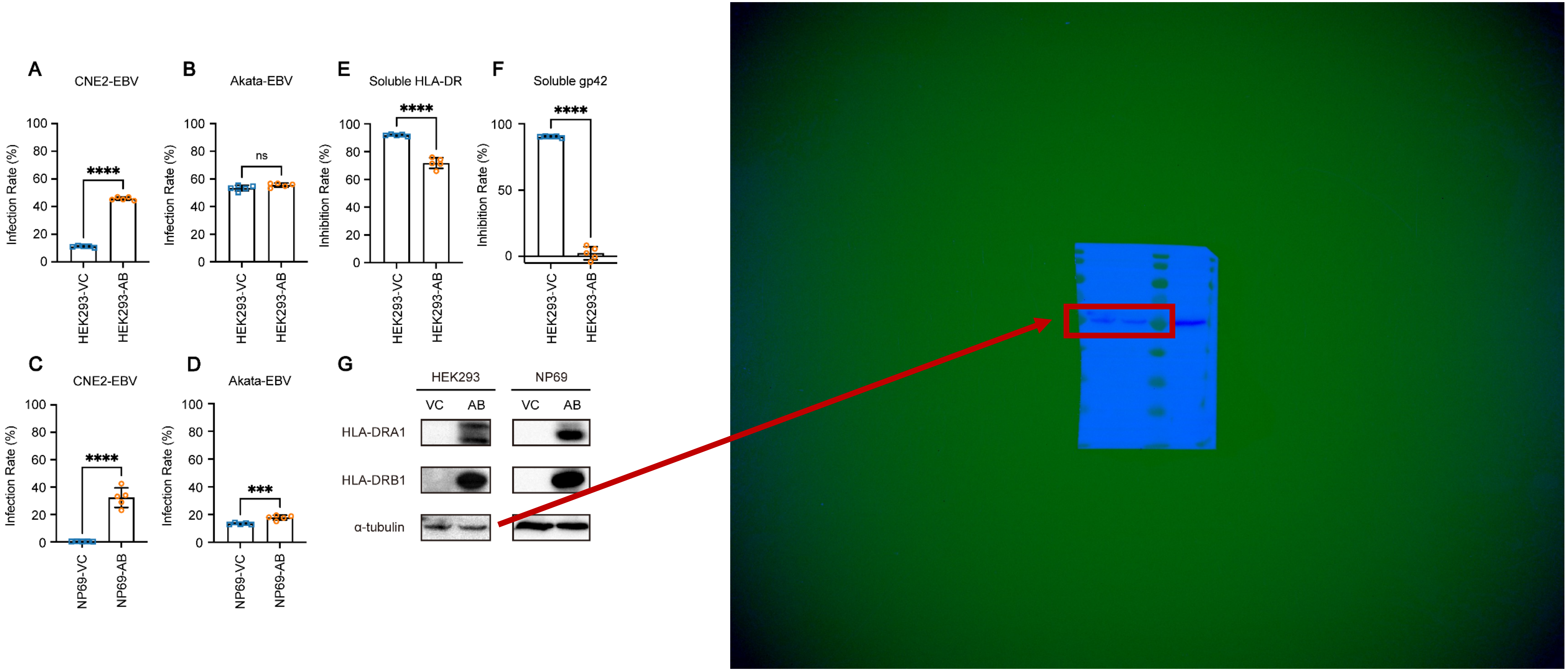

Full unedited gel for Figure 4G. Anti-α-tubulin (2125S, CST) and anti-rabbit IgG-HRP (31460, Thermo Fisher) were used for blotting.

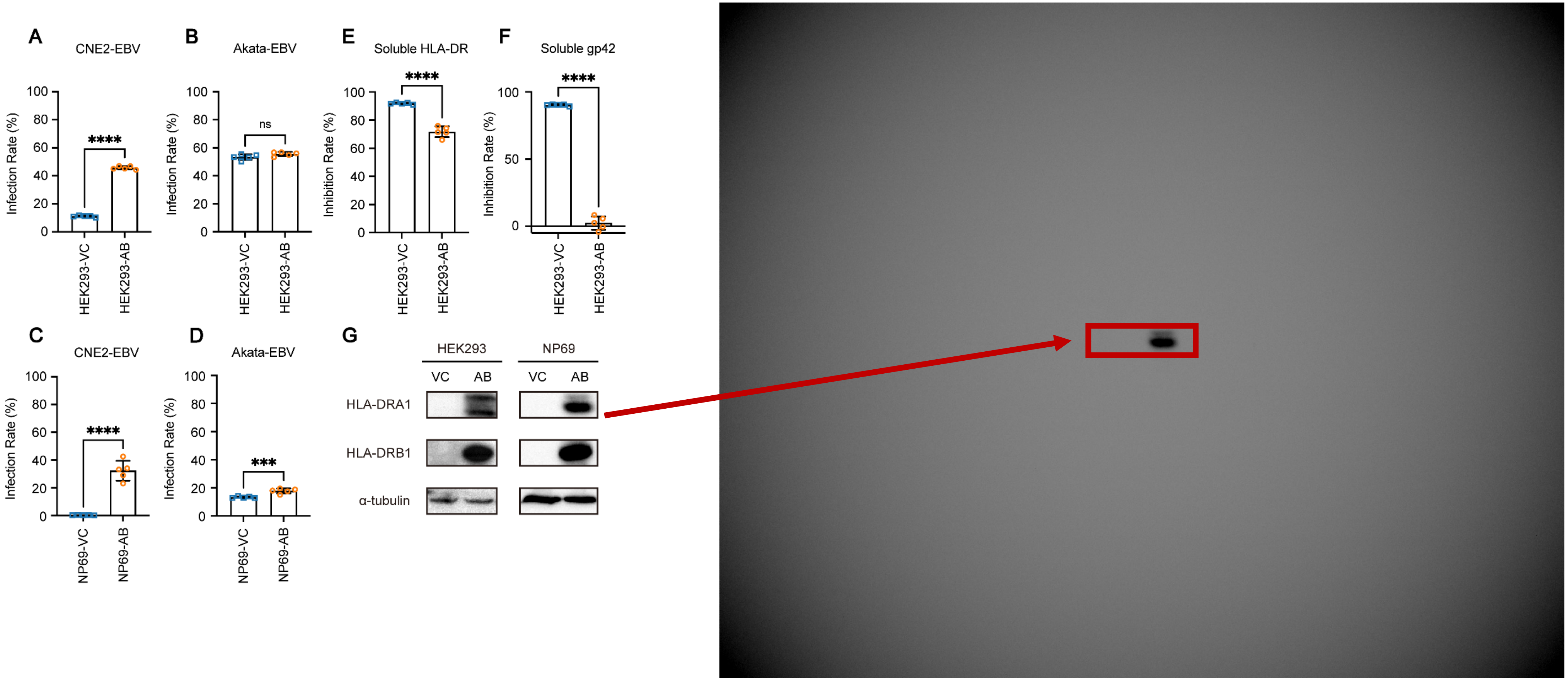

Full unedited gel for Figure 4G. Anti-HLA-DRA1 (ab92511, Abcam) and anti-rabbit IgG-HRP (31460, Thermo Fisher) were used for blotting.

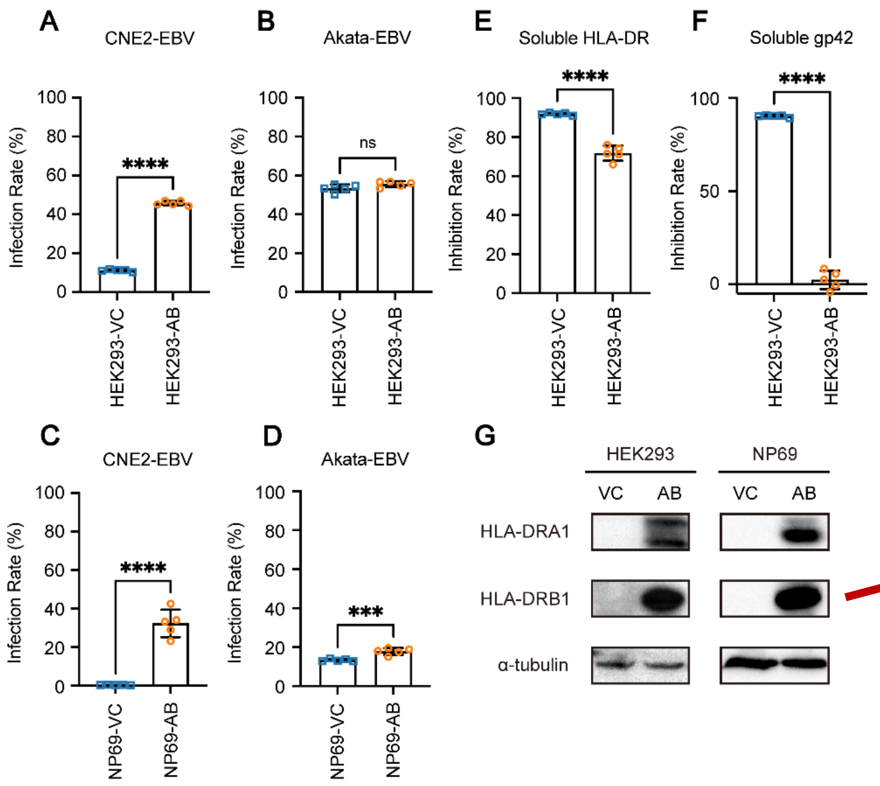

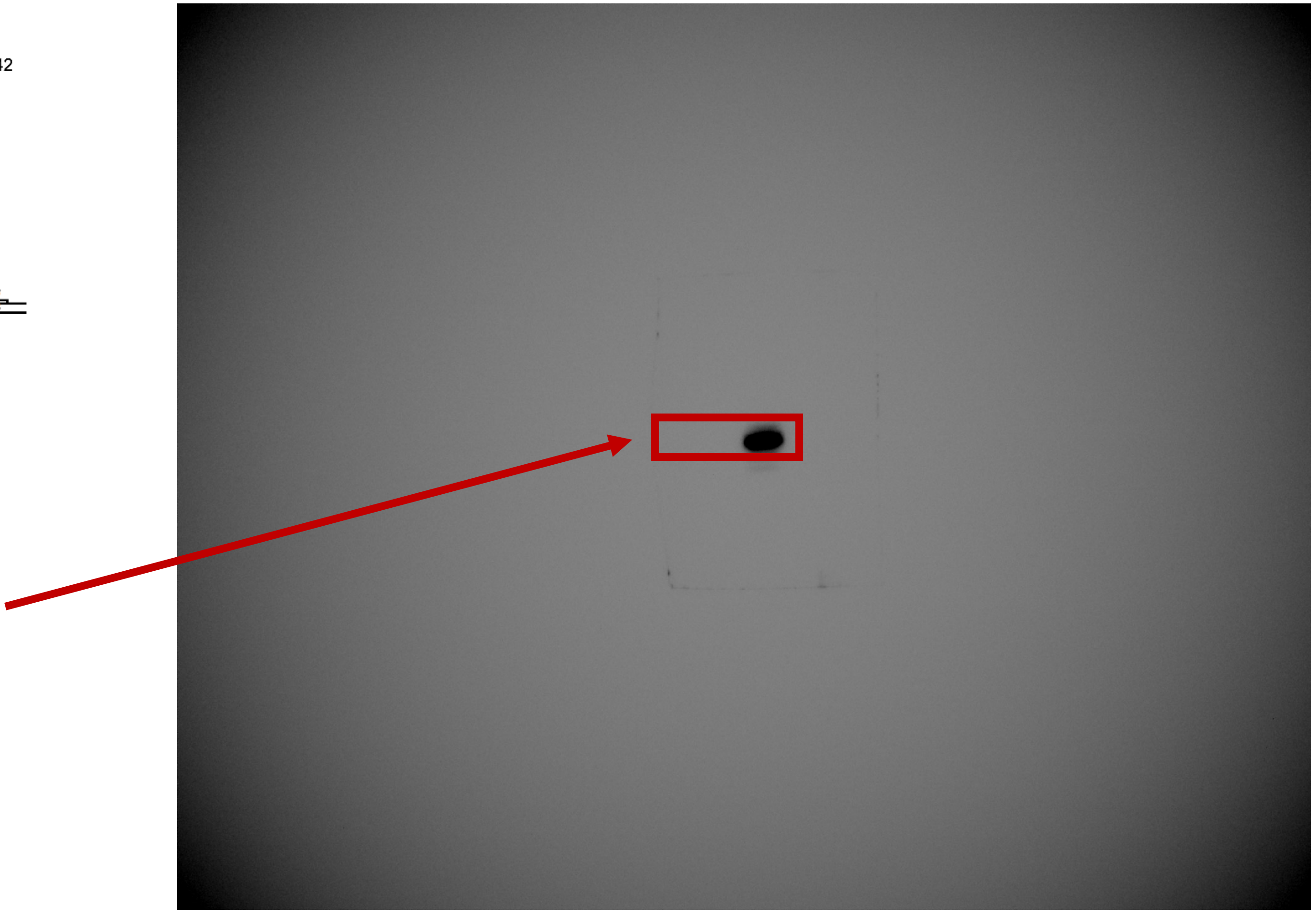

Full unedited gel for Figure 4G. Anti-HLA-DRB1 (ab133578, Abcam) and anti-rabbit IgG-HRP (31460, Thermo Fisher) were used for blotting.

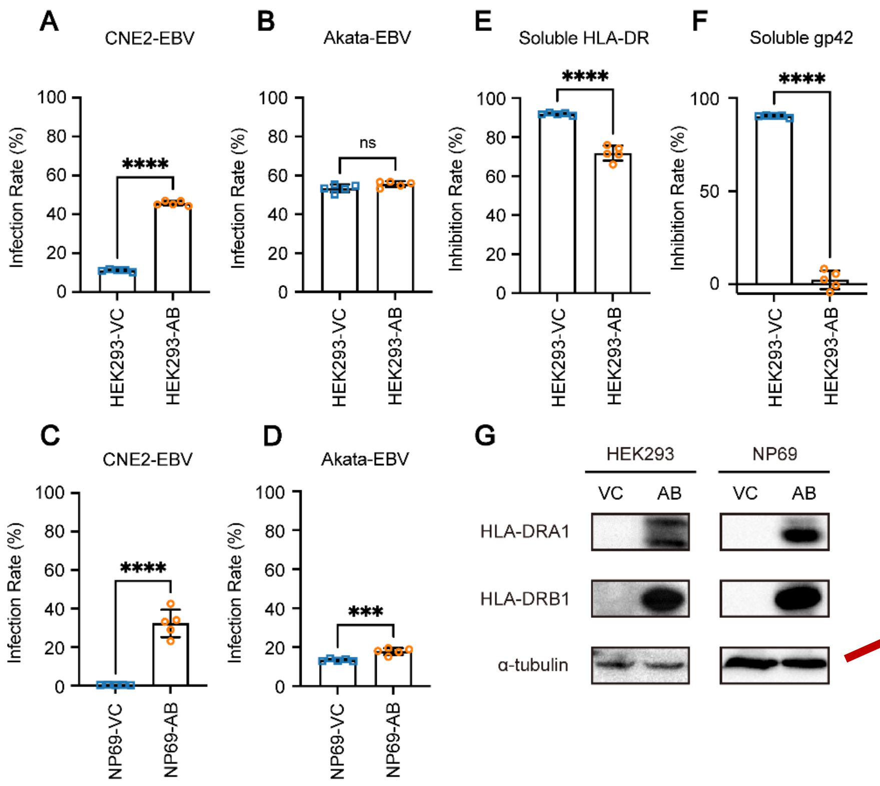

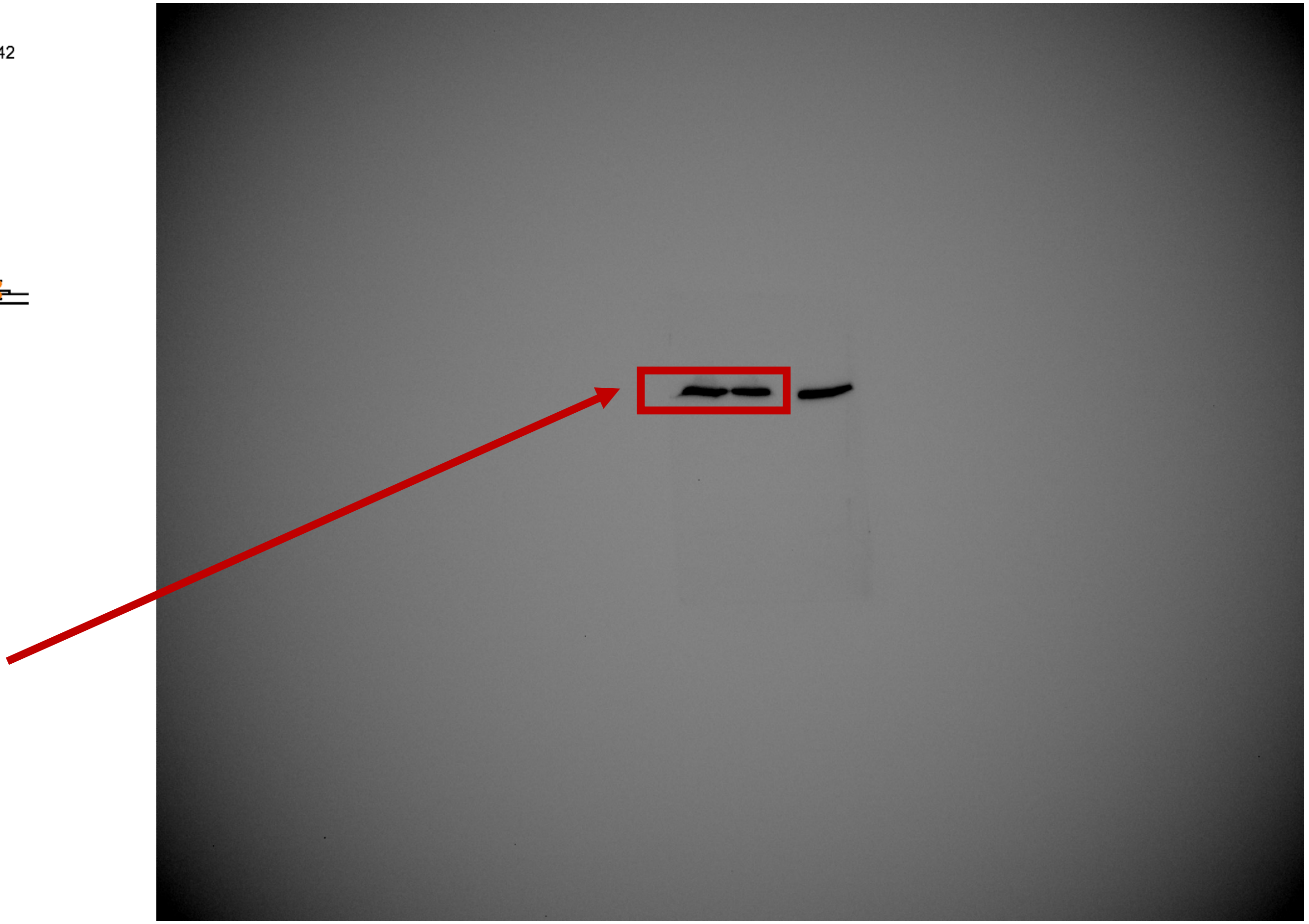

Full unedited gel for Figure 4G. Anti-α-tubulin (2125S, CST) and anti-rabbit IgG-HRP (31460, Thermo Fisher) were used for blotting.
